# Non-tuberculous mycobacterial pulmonary diseases in France: an 8 years nationwide study

**DOI:** 10.1186/s12879-021-06825-x

**Published:** 2021-11-17

**Authors:** Nicolas Veziris, Claire Andréjak, Stéphane Bouée, Corinne Emery, Marko Obradovic, Raphaël Chiron

**Affiliations:** 1 Sorbonne Université, Centre d’Immunologie et des Maladies Infectieuses (Cimi-Paris), UMR 1135, Département de Bactériologie, Hôpital Saint-Antoine, Centre National de Référence des Mycobactéries, APHP.Sorbonne Université, Paris, France; 2 EA4294 AGIR, Université Picardie Jules Verne, 80000 Amiens, France; 3 Service de Pneumologie, CHU Amiens, 1 Rue du Professeur Christian Cabrol, 80000 Amiens, France; 4 RWE, CEMKA, 43 Bd Maréchal Joffre, 92340 Bourg La Reine, France; 5 Insmed Germany GmbH, The Squaire 12 Am, Flughafen, 60549 Frankfurt, Germany; 6 HydroSciences Montpellier, CNRS, IRD, Univ Montpellier, 15, avenue Charles Flahault, 34093 Montpellier, France; 7 University Hospital Centre Montpellier, CF center, 371 Av. du Doyen Gaston Giraud, 34090 Montpellier, France

**Keywords:** Non-tuberculous mycobacteria, Lung infection, Antibiotics, Mortality, Real world evidence, Claim database

## Abstract

**Background:**

The objective of the study was to describe the epidemiology, management and cost of non-tuberculous mycobacteria pulmonary disease (NTM-PD) in France.

**Methods:**

A retrospective analysis was performed using the SNDS *(“Système national des données de santé”*) database over 2010–2017. Patients with NTM-PD were identified based on the ICD10 codes during hospitalizations and/or specific antibiotics treatment regimens. The study population was matched (age, sex and region) to a control group (1:3) without NTM-PD.

**Results:**

5628 patients with NTM-PD (men: 52.9%, mean age = 60.9 years) were identified over the study period and 1433 (25.5%) were treated with antibiotics. The proportion of patients still receiving treatment at 6 and 12 months was 40% and 22%, respectively. The prevalence of NTM-PD was estimated at 5.92 per 100,000 inhabitants and the incidence rate of NTM-PD remained stable over time between 1.025/100,000 in 2010 and 1.096/100,000 in 2017. Patients with NTM-PD had more co-morbidities compared to controls: corticoids (57.3% vs. 33.8%), chronic lower respiratory disease (34.4% vs. 2.7%), other infectious pneumonia (24.4% vs. 1.4%), malnutrition (based on hospitalization with the ICD-10 code reported during a hospital stay as a main or secondary diagnosis) (22.0% vs. 2.0%), history of tuberculosis (14.1% vs. 0.1%), HIV (8.7% vs. 0.2%), lung cancer and lung graft (5.7% vs. 0.4%), cystic fibrosis (3.2% vs. 0.0%), gastro-esophageal reflux disease (2.9% vs. 0.9%) and bone marrow transplant (1.3% vs. 0.0%) (p < 0.0001). The mean Charlson comorbidity index score was 1.6 (vs. 0.2 for controls; p < 0.0001). NTM-PD was independently associated with an increased mortality rate with a hazard ratio of 2.8 (95% CI: 2.53; 3.11). Mortality was lower for patients treated with antibiotics compared to untreated patients (HR = 0.772 (95% CI [0.628; 0.949]). Annual total expenses the year following the infection in a societal perspective were € 24,083 (SD: 29,358) in NTM-PD subjects vs. € 3402 (SD: 8575) in controls (p < 0.0001). Main driver of the total expense for NTM-PD patients was hospital expense (> 50% of the total expense).

**Conclusion:**

Patients with NTM-PD in France were shown to have many comorbidities, their mortality risk is high and mainly driven by NTM-PD, and their management costly. Only a minority of patients got treated with antibiotics and of those patients treated, many stopped their therapy prematurely. These results underline the high burden associated with NTM-PD and the need for improvement of NTM-PD management in France.

**Supplementary Information:**

The online version contains supplementary material available at 10.1186/s12879-021-06825-x.

## Background

More than 190 species and subspecies of non-tuberculous mycobacteria (NTM) have been described. Among these, some like *Mycobacterium avium* complex (MAC), may cause pulmonary disease (NTM-PD) with increasing frequency in non-acquired immunodeficiency syndrome (AIDS) patients [1, 2]. The incidence and prevalence of NTM-PD has even surpassed that of tuberculosis in some settings [3, 4]. This increase has been observed in older individuals and those with underlying bronchiectasis and may be associated with multifactorial factors. In Europe, it is estimated that the annual prevalence rate of diagnosed NTM-PD cases is in the range of 3–6/100,000 [4–6]. In France studies on NTM-PD have been conducted either based on cystic fibrosis registries or from laboratory databases from university hospitals [7] but not nationally generalizeable estimates.

The economic burden of NTM-PD in France is expected to be substantial due to its challenging management with frequent hospitalizations and the need for long-term antibiotic treatment. In Germany, NTM-PD mean direct expenditure was estimated to be nearly fourfold that of matched controls [8] and hospitalizations accounted for 63% of the total costs. Similar costs for NTM-PD have been observed in other western countries [9]. However, estimates of treatment costs for patients with newly diagnosed NTM-PD are currently not available in France. Therefore, we aimed to describe the epidemiology, comorbidities, management, mortality, and costs of NTM-PD in a real world setting in France.

## Methods

### Study design

We conducted a retrospective study using the French nationwide claims and hospitalisation database, the Système National des Données de Santé (SNDS) which covers more than 99% of the French population (nearly 66 million people) and represents one of the largest medico-administrative databases in the world. The SNDS database contains anonymous data on healthcare encounters (public and private), diagnostic-related groups, drugs, medical devices, procedures, laboratory tests (without results), date of death excluding cause, hospitalizations with ICD10 codes and discharge summaries with the main and secondary diagnoses.

Patients with NTM-PD were identified in the database based on hospitalizations with ICD-10 codes specific for NTM-PD infection and/or antibiotics combination for the treatment of the disease using outpatient drug consumptions. The detailed algorithm is reported in the additional files (Additional file 1: Tables S1–S3).

Newly diagnosed patients with NTM-PD (adults and children) not previously treated or hospitalized for NTM-PD in the last 3 years were included in the study from January 01/2010 to December 31/2017.

Socio-demographic characteristics included age, sex, residence, supplementary universal health care coverage (CMUc), status and registration for long-term disease with ICD-10 codes, and date of death, if any. Patients with NTM-PD were matched on age, sex, and region of residence with controls who were not selected by the algorithm to identify NTM-PD patients, with a ratio of 1:3. Risk factors for NTM-PD (including malnutrition) were identified through specific ICD-10 codes for long-term diseases, hospitalizations, and treatment in the past 3 years prior to NTM-PD diagnosis. Healthcare consumptions and costs related to hospitalizations and outpatient visits were also assessed.

An economic analysis was conducted and involved patients who were newly diagnosed with NTM-PD between January 01/2012 and December 31/2016; healthcare resource use and costs over one-year period following the diagnosis of NTM-PD were analyzed. The economic evaluation was assessed from the French Public Health Insurance perspective, with a separate analysis that also included patient out of pocket costs for services or drugs that are only partially reimbursed.

Outpatient costs that were analyzed relating to medical honoraria, dental fees, pharmacy and other products, laboratory tests, paramedical interventions, medical devices, transportation, and other cares. Indirect costs due to daily allowance for sick leave are reported separately.

### Statistical analysis

Mean, standard deviation, median, minimum, and maximum were used to describe quantitative variables and percentages for qualitative variables. Bivariate analyses were performed and the Chi^2^ test was used to assess qualitative variables, whereas the Yates continuity correction or Fisher’s exact test evaluated sample sizes less than 5. For quantitative variables, a Student’s t-test or analysis of variance was performed when distribution was close to normal; otherwise, non-parametric tests including Wilcoxon and Kruskal–Wallis were used. Survival was analyzed with Kaplan–Meier curves and log rank test with December 31/2017 as the time point for censored observation. Cox multivariate model adjusted for age, sex, residence and comorbidities was used to compare: (1) NTM-PD patients to the controls and (2) NTM-PD patients treated with antibiotics compared to untreated NTM-PD patients.

## Results

A total of 5628 patients with NTM-PD were identified in the SNDS database between 2010 and 2017, 4898 patients from hospitalizations and 730 from outpatient drug consumptions. Among patients identified through hospitalizations, 703 had a specific treatment for NTM-PD vs. 4195 with no treatment. The mean age was 60.9 years (SD ± 19.5) and 52.9% were males. The Universal Health Coverage for low-income individuals was observed in 8.8% of patients (Table 1).

**Table 1:** Patient characteristics

	Treated patients with NTM-PD n (%)	Untreated patients with NTM-PD n (%)	Total n (%)	p-value
Incident population	1433 (25.5)	4195 (74.5)	5628 (100)	
Mean age (SD) at the time of diagnosis (T0)	56.7 (17.4)	62.3 (20.0)	60.9 (19.5)	< 0.0001
Age at T0 in groups				
< 10 years	3 (0.2)	92 (2.2)	95 (1.7)	< 0.0001
10–19 years	40 (2.8)	92 (2.2)	132 (2.3)	
20–29 years	75 (5.2)	158 (3.8)	233 (4.1)	
30–39 years	133 (9.3)	200 (4.8)	333 (5.9)	
40–49 years	204 (14.3)	363 (8.7)	567 (10.1)	
50–59 years	277 (19.4)	661 (15.8)	938 (16.7)	
60–69 years	336 (23.5)	884 (21.1)	1220 (21.7)	
70–79 years	244 (17.1)	905 (21.6)	1149 (20.4)	
80–89 years	112 (7.8)	712 (17.0)	824 (14.7)	
≥ 90 years	6 (0.4)	127 (3.0)	133 (2.4)	
Sex				
Male	733 (51.2)	2245 (53.5)	2978 (52.9)	0.1216
Female	700 (48.8)	1950 (46.5)	2650 (47.1)	
Universal Health Coverage				
No	1292 (90.2)	3843 (91.6)	5135 (91.2)	0.0940
Yes	141 (9.8)	352 (8.4)	493 (8.8)	

*SD* standard deviation, *T0* time of diagnosis

**Fig. 1:**
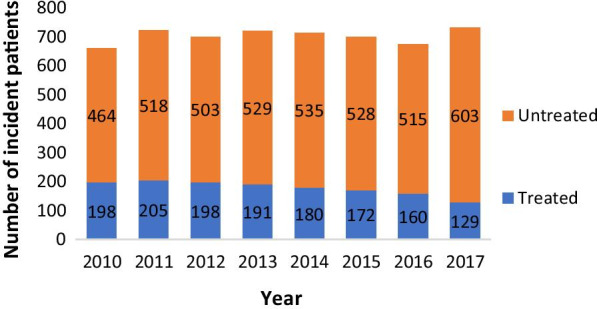
Number of NTM-PD incidence cases from 2010 to 2017

A total of 3954 patients were diagnosed with NTM-PD between January 01/2010 and December 31/2017; thus, the prevalence of NTM-PD was estimated at 5.92 per 100,000 inhabitants over 8 years. The incidence rate of NTM-PD (Fig. 1) remained stable over time, with a min of 1.025/100,000 in 2010 (N = 662) and a max of 1.096/100,000 (N = 732) in 2017 with slight variations in-between years.

Matched controls could not be identified for all patients in the study; therefore, comparisons included 4447 NTM-PD cases matched to 13,341 controls for age, sex, and region.

Analysis of comorbidities (Table 2) found a significantly higher Charlson [10] Comorbidity Index (CCI) mean score in patients with NTM-PD (1.6) compared to controls (0.2) (p < 0.0001). However, there was no difference between treated and untreated groups on the CCI score (p = 0.3). Overall, patients with NTM-PD had a higher proportion of risk factors compared to controls including respectively corticoid treatment in the last 3 years (57.3% vs. 33.8%, p < 0.0001), chronic lower respiratory disease (34.4% vs. 2.7%, p < 0.0001), other infectious pneumonia (24.4% vs. 1.4%, p < 0.0001), malnutrition (22.0% vs. 2.0%, p < 0.0001), and history of tuberculosis (14.1% vs. 0.1%, p < 0.0001).

**Table 2:** Risk factors for NTM-PD patients and matched controls

	Cases n (%)	Controls n (%)	p-value cases/controls	Treated NTM-PD n (%)	Untreated NTM-PD n (%)	p-value treated/not treated
Incident population	4447 (25.0)	13,341 (75.0)		1433 (25.5)	4195 (74.5)	
Charlson comorbidity index score (SD)	1.6 (2.5)	0.2 (0.9)	< 0.0001	1.7 (2.7)	1.5 (2.4)	0.2907
Risk factors before NTM-PD diagnosis						
Chronic lower respiratory diseases	1529 (34.4)	359 (2.7)	< 0.0001	428 (29.9)	1,547 (36.9)	< 0.0001
COPD (J44)	836 (18.8)	168 (1.3)	< 0.0001	203 (14.2)	887 (21.1)	< 0.0001
Bronchiectasis (J47)	472 (10.6)	16 (0.1)	< 0.0001	123 (8.6)	486 (11.6)	0.0016
Other lower respiratory Chronic diseases	927 (20.8)	245 (1.8)	< 0.0001	277 (19.3)	911 (21.7)	0.0560
HIV infection	389 (8.7)	30 (0.2)	< 0.0001	212 (14.8)	211 (5.0)	< 0.0001
Lung cancer and lung graft	252 (5.7)	55 (0.4)	< 0.0001	52 (3.6)	272 (6.5)	< 0.0001
Bone marrow transplant	60 (1.3)	3 (0.0)	< 0.0001	17 (1.2)	49 (1.2)	0.9558
Gastro-esophageal reflux disease	131 (2.9)	120 (0.9)	< 0.0001	44 (3.1)	121 (2.9)	0.7184
Smoking	164 (3.7)	112 (0.8)	< 0.0001	52 (3.6)	146 (3.5)	0.7923
Other infectious pneumonia	1085 (24.4)	184 (1.4)	< 0.0001	286 (20.0)	1107 (26.4)	< 0.0001
Tuberculosis	628 (14.1)	14 (0.1)	< 0.0001			
Malnutrition	978 (22.0)	261 (2.0)	< 0.0001			
Corticoids	2550 (57.3)	4504 (33.8)	< 0.0001			
Cystic fibrosis	143 (3.2)	1 (0.0)	< 0.0001			

*SD* standard deviation

Even if statistically significant, no notable differences between the treated and untreated group were found, except higher rates of HIV infection in treated vs. untreated patients (14.8% vs. 5%).

### Mortality

**Fig. 2:**
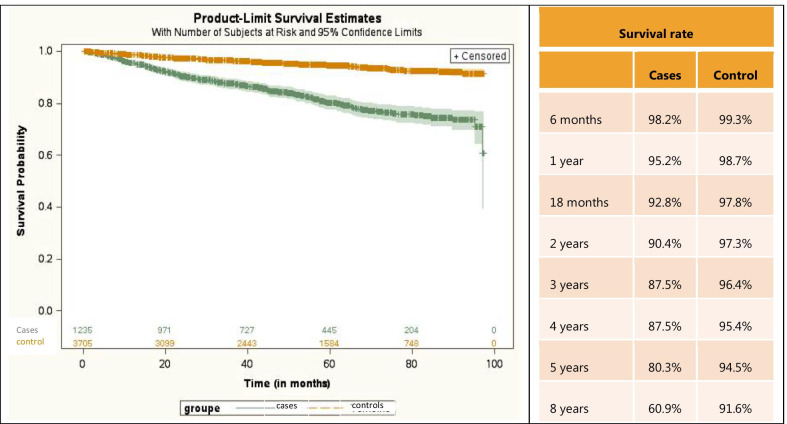
Kaplan Meier survival curves in NTM-PD patients and matched controls

A first analysis showed that the mortality of 4447 NTM-PD cases was significantly higher than that of 13,341 controls (p < 0.0001) (Fig. 2).

In multivariate analysis (Table 3), after adjustment for age, residence, sex,, and risk factors, the risk of mortality was more than 2 times higher in NTM-PD patients compared to controls (HR = 2.804 (95% CI [2.532; 3.104]). The risk of mortality was more likely to increase in older patients with NTM-PD including those aged 70–79 years (HR = 16.081, 95% CI: 6.646–38.908), 80–89 years (HR = 33.654, 95% CI: 13.910–81.423), and more than 90 years (HR = 56.860, 95 CI: 23.209–139.303). Mortality was also higher in patients with Universal Health Coverage, HIV infection, lung cancer and lung graft, other infectious pneumonia, malnutrition, and other chronic obstructive pulmonary disease. Mortality was lower in female patients vs. males and in patients who had mucopurulent chronic bronchitis and bronchiectasis.

**Table 3:** Cox multivariate analysis for mortality in NTM-PD patients and matched controls

Covariates		Hazard ratio	95% CI	
NTM-PD case/matched controls (Reference: controls)	NTM-PD case/matched controls	2.804	2.532	3.104
Age (reference < 10 years)	10–19 years	0.818	0.244	2.740
	20–29 years	1.571	0.589	4.192
	30–39 years	2.283	0.903	5.772
	40–49 years	2.704	1.093	6.688
	50–59 years	5.736	2.360	13.937
	60–69 years	9.204	3.800	22.290
	70–79 years	16.081	6.646	38.908
	80–89 years	33.654	13.910	81.423
	≥ 90 years	56.860	23.209	139.303
Sex (reference: males)	Female	0.668	0.612	0.730
Universal Health Coverage	yes	1.423	1.172	1.728
HIV infection	Yes	1.592	1.244	2.038
Lung cancer and graft	Yes	2.492	2.097	2.962
Other infectious pneumonia	Yes	1.353	1.202	1.524
Tuberculosis	Yes	0.904	0.773	1.058
Malnutrition	Yes	2.042	1.821	2.290
Corticoids	Yes	1.040	0.952	1.137
Cystic fibrosis	Yes	1.508	0.774	2.936
Mucopurulent chronic bronchitis	Yes	0.726	0.549	0.958
Unspecified chronic bronchitis	Yes	1.227	1.023	1.472
Emphysema	Yes	1.080	0.904	1.290
Other chronic obstructive pulmonary disease	Yes	1.651	1.451	1.879
Bronchiectasis	Yes	0.713	0.591	0.861

In a second multivariate analysis performed on the NTM-PD cohort and adjusted for age, residence, sex, and comorbidities, the risk of mortality was lower for treated patients with antibiotics compared to untreated patients (HR = 0.776 (95% CI [0.628; 0.949]).

### Treatments

Out of the 5628 patients, 25.5% (1433) had received antibiotics to treat NTM-PD vs. 74.5% (4195) with no treatment (Table 4).

**Table 4:** Antibiotic treatments for NTM-PD patients

	Hospitalization with an NTM-PD ICD-10 code (N =5628 ) n (%)		Total
	Yes N = 4898	No N = 730	
Treatment			
Any antibiotics	703 (14.4)	730 (100)	1433 (25.5)
Clarithromycin + Ethambutol	232 (33.0)	275 (377)	507 (34.4)
Clarithromycin + Rifampin + Ethambutol	159 (22.6)	166 (22.7)	325 (22.1)
Clarithromycin + Rifampin	31 (4.4)	121 (16.6)	152 (10.3)
Clarithromycin	137 (19.5)	–	137 (9.3)
Azithromycin + Ethambutol	43 (6.1)	28 (3.8)	71 (4.8)
Azithromycin + Rifampin	4 (0.6)	64 (8.8)	68 (4.6)
Moxifloxacin + Rifampin + Ethambutol	23 (3.3)	36 (4.9)	59 (4.0)
Azithromycin + Rifampin + Ethambutol	18 (2.6)	25 (3.4)	43 (2.9)
Moxifloxacin + Ethambutol	17 (2.4)	–	17 (1.2)
Clarithromycin + Moxifloxacin + Ethambutol	11 (1.6)	4 (0.5)	15 (1.0)
Clarithromycin + Moxifloxacin	7 (1.0)	–	7 (0.5)
Clarithromycin + Ethambutol + Amikacin	5 (0.7)	1 (0.1)	6 (0.4)
Clarithromycin + Amikacin	4 (0.6)	1 (0.1)	5 (0.3)
Clarithromycin + Moxifloxacin + Rifampin	1 (0.1)	3 (0.4)	4 (0.3)
Moxifloxacin + Ethambutol + Amikacin	2 (0.3)	1 (0.1)	3 (0.2)
Azithromycin + Amikacin	1 (0.1)	1 (0.1)	2 (0.1)
Azithromycin + Rifampin + Ethambutol + Amikacin	1 (0.1)	1 (0.1)	2 (0.1)
Clarithromycin + Ethambutol + Rifabutin	1 (0.1)	1 (0.1)	2 (0.1)
Clarithromycin + Rifampin + Ethambutol + Amikacin	2 (0.3)	–	2 (0.1)
Azithromycin + Amikacin + Ethambutol	–	1 (0.1)	1 (0.1)
Clarithromycin + Azithromycin + Rifampin + Ethambutol	1 (0.1)	–	1 (0.1)
Clarithromycin + Rifampin + Ethambutol + Rifabutin	–	1 (0.1)	1 (0.1)
Moxifloxacin + Amikacin	1 (0.1)	–	1 (0.1)
Moxifloxacin + Azithromycin	1 (0.1)	–	1 (0.1)
Moxifloxacin + Rifabutin	1 (0.1)	–	1 (0.1)

Among the 4,898 hospitalized patients, 703 (14.4%) had a specific treatment for NTM and 4195 (85.6%) did not receive any treatment. The most frequently used treatment first line regimens were: Clarithromycin + Ethambutol for 34.4% of patients, Clarithromycin + Rifampin + Ethambutol (22.1%), Clarithromycin + Rifampin (10.3%), and Clarithromycin monotherapy (9.3%). Other combinations represented less than 5% of patients for each of them (Table 4).

**Fig. 3:**
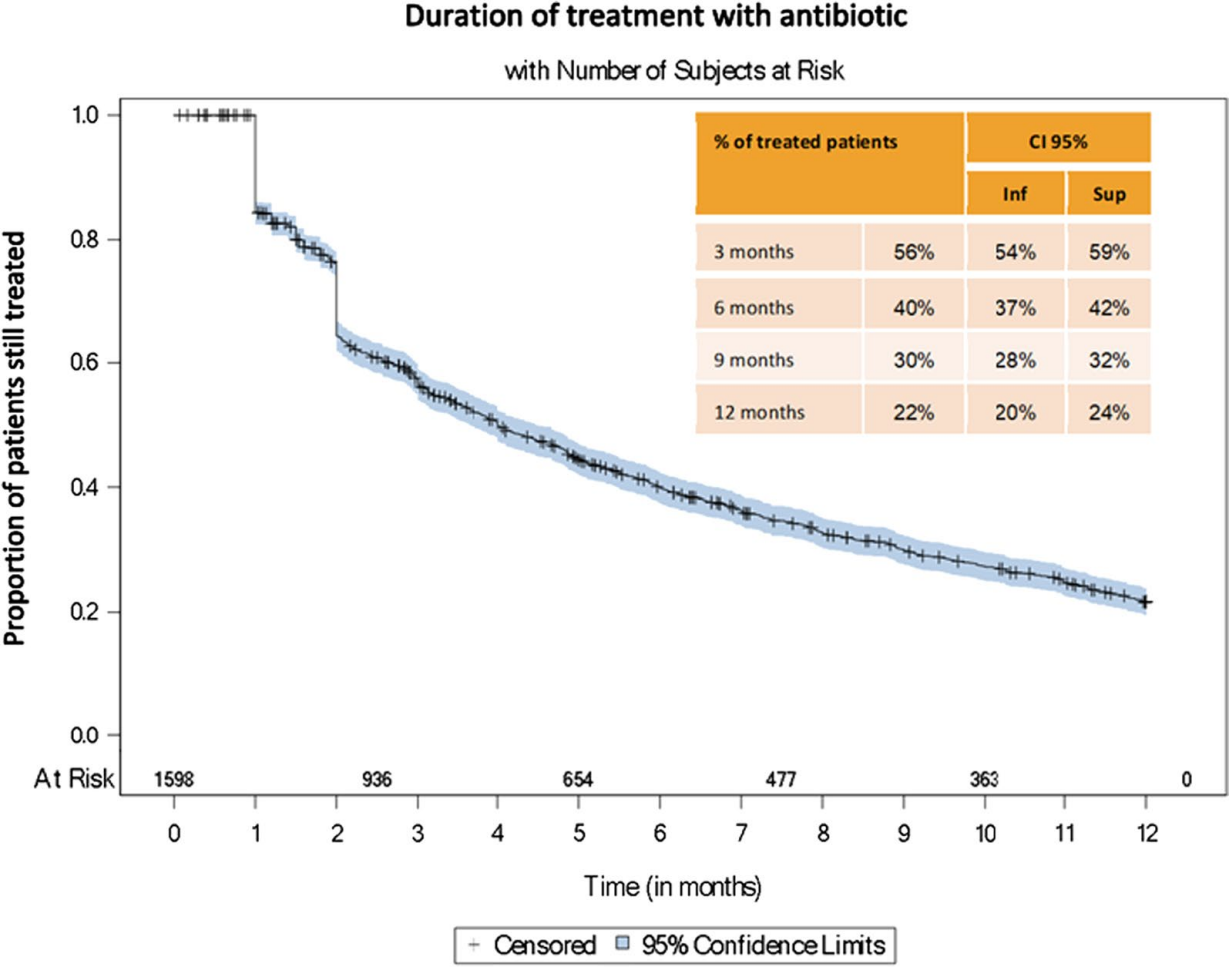
Kaplan Meier survival curves for treatment duration in NTM-PD patients

Figure 3 depicts the proportion of patients maintaining treatments at 3, 6, 9, and 12 months. Half of the patients (56%) were still treated at 3 months, 40% at 6 months, 30% at 9 months, and only 22% at 12 months. The majority of patients discontinued before 12 months of treatment.

### Healthcare expenditures

This analysis included 3950 cases and 11,850 controls. A total of 3,641 NTM-PD patients (92.2%) had been hospitalized at least once since diagnosis compared to 2484 controls (21.0%). For those hospitalized at least once, the length of hospital stay was 40.3 days for cases vs. 16.5 days for controls; mean hospital stay per patient was 5.0 for cases and 2.7 for controls.

### Economic analysis

A total of 2,683 cases and 8,049 controls were included in the economic analysis.

The mean total cost reimbursed in the year following NTM-PD diagnosis was significantly higher for cases compared to controls, €22,966 vs. €2709, respectively (p < 0.0001). Total expenses (societal perspective) followed the same trend with a greater amount for cases than controls, €24,083 vs. €3402, respectively (p < 0.0001).

**Fig. 4:**
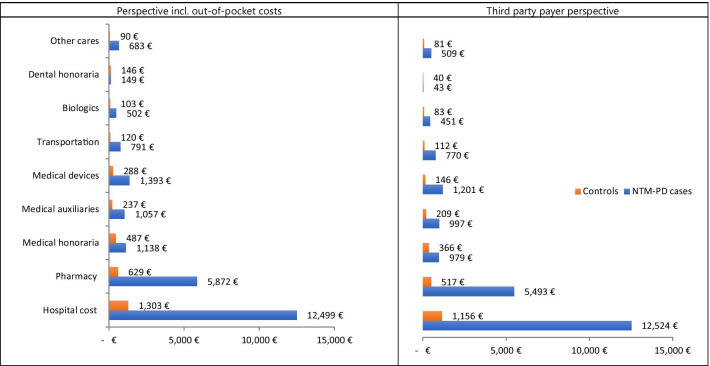
Healthcare Costs in NTM-PD cases and controls. All differences between NTM-PD cases and controls are statistically significative (p<0.0001) except for dental honoraria

From the French Public Health Insurance perspective (Fig. 4) the mean cost for hospitalizations in the year following NTM-PD diagnosis was €12,524 for cases vs. €1156 for controls (p < 0.0001). The total cost for outpatient care in the year following NTM-PD diagnosis was €10,442 for NTM-PD patients and €1553 for controls. Costs of drugs were estimated at €5493 for cases (52.6% of the total amount) vs. €517 for controls (33% of the total amount).

From a perspective incl. out-of-pocket costs (Fig. 4), the mean cost for hospitalizations in the year following NTM-PD diagnosis was €12,499 for cases vs. €1303 for controls (p < 0.0001). The total cost of outpatient care in the year following NTM-PD diagnosis was €11,584 for NTM-PD patients and €2099 for controls. Costs of drugs were estimated at €5872 for cases (50.6% of the total amount) vs. € 629 for controls (30% of the total amount).

## Discussion

This retrospective study using the SNDS, a French nationwide claims and hospitalisation database, described the epidemiology, comorbidities, management, mortality, and costs of NTM-PD in a real world setting in France. Thanks to its comprehensive coverage of the whole population (99%), the SNDS database allowed this study to include all NTM-PD patients identified with the ICD-10 codes in France. Our study not only has demonstrated the higher mortality rate in NTM-PD patients and the economic burden related to this condition but also suggested that guidelines are not always respected.

Prevalence (6/100,000 inhabitants over 8 years) and incidence (1/100,000) were close to those reported in previous studies conducted in Europe with same or different methods including Delphi method, registry analysis, retrospective study of laboratory data [5, 8, 11–13]. These converging data suggest that the true incidence and prevalence must be close to those we found. Whether incidence of NTM-PD is increasing or is better diagnosed or reported is debated in the literature. The present study over 8 years did not show any increase of incidence which was close to the one reported in France 15 years ago [13].

Surprisingly, the proportion of men and women was comparable in our study in contrast to the higher proportion of women found in previous studies. As we did not include NTM-PD cases that were both not treated and not hospitalized, we may have missed women who outlived men and were followed by watchful waiting. This could explain the lower ratio of women we observed compared to previous studies. It should be noted that the same proportion of men and women, as compared to our study, was also found in Germany [11] and Denmark [12].

Among the different factors associated with NTM-PD that can be prevented, malnutrition was 10 times more frequent in NTM-PD patients than in controls. Weight loss has been reported as being a negative prognostic factor for NTM-PD [14]. Therefore, patients’ nutritional status should be assessed by a dietician particularly in those with NTM-PD cumulative risk factors.

We found that NTM-PD patients had a twice higher risk of mortality than controls in line with previous studies conducted in Germany and in the US [8, 18]. After 8 years there was a 30% difference in mortality between NTM-PD patients and controls. The 10 years survival rate predicted by Charlson index [10] for NTM-PD cases was between 90 and 95% whereas we found 61% after 8 years of follow up, underlying the impact of NTM-PD on mortality.

We observed that advanced age was associated with a greater risk of mortality. Interestingly, elderly patients were less likely to be treated in our study. This shows the reluctance of healthcare providers to initiate long-term and complex regimens in older patients who may be already treated for other comorbid conditions with multitude treatments. However, given this increasing mortality our data rather support treatment initiation in line with recent recommendations [15]. This is further supported by the survival analysis that showed that treated patients had 23% reduced risk of dying compared to untreated after controlling for confounding factors.

In a study conducted on a Danish registry [12], predictors of death were age ≥ 65 years (HR: 9.17) and male sex (female sex HR, 0.73). This is consistent with our study which shows a decreased risk of mortality in female NTM-PD patients. NTM-PD in women tend to manifest with bronchiectasis and has better prognosis than patients, usually men, who have the cavitary form of the disease [16]. Indeed, our study suggests a lower risk of mortality in NTM-PD patients with bronchiectasis compared to NTM-PD patients without bronchiectasis (HR: 0.713, 95% CI [0.591–0.861]). We did not find a significant association between emphysema and the risk of mortality in NTM-PD patients; however, those with other COPD were more likely to die compared to those without other COPD. A higher rate of mortality in NTM-PD patients with COPD has been consistently reported and accounted for 41.5% over the period of 39 months compared to 22.4% for the overall NTM-PD group, including those with and without COPD [8].

In our study, 85.6% of patients identified through hospitalizations did not receive any antibiotics to treat NTM-PD. However, in those treated for NTM-PD, clarithromycin monotherapy was observed in 19.5% of patients. This is particularly alarming since clarithromycin resistance has been reported in 16% of NTM-PD patients treated with clarithromycin monotherapy and this resistance that is prevented by multidrug therapy is associated with a poor prognosis [17, 18]. Also worrying was the rapid decline of the proportion of patients on treatment reaching 30% at 9 months whereas all recommended regimens last at least on year [16]. Together with increased long-term mortality, these data on poor treatment management underline the need for implementation of educational measures in France.

In our study, total expenses for cases were sevenfold higher than that of controls, €24,083 and €3402, respectively (p < 0.0001). Total costs were driven by hospitalizations followed by drugs, and finally outpatient care. Our results are consistent with previous studies reporting a higher cost in NTM-PD patients compared to matched-controls. In Germany, Diel et al. [8] estimated the mean direct expenditure per NTM-PD patient to be €39,559.60 over a period of 39 months which was almost fourfold that of matched control (€10, 006.71). Furthermore, NTM-PD patients were three times more likely to be hospitalized than controls, and hospitalizations represented 63% of the total costs. Goring et al. [9] conducted a study in a cohort of MAC patients refractory to treatment and estimated annual NTM-PD-related costs at $16,209 in Canada, €11,626 in Germany, €17,881 in France, and £9727 in the UK. Marras et al. [19] reported higher healthcare expenditures in newly diagnosed NTM-PD patients than in controls the first year ($72,475 vs. $28,405) and second year ($48,114 vs. $28,990), respectively. Interestingly, cost for an NTM-PD patient clearly surpasses the annual cost per patient for chronic respiratory diseases including COPD (€1013) or asthma (€1950) among others [20].

The limitations of the SNDS database are inherent to those of claims databases. The paucity of clinical information and laboratory results did not allow the use of the ATS/IDSA guidelines which include clinical, radiologic, and bacteriologic criteria to pose a diagnosis of NTM-PD [2]. We also did not have information on specific NTM species to be able to have species-specific results. Identification of NTM-PD cases using the ICD-10 codes along with the prescription of antimycobacterial treatments may have covered the majority of patients with NTM-PD; however, we may have missed patients who may have been misclassified with the ICD-10 codes. A better specificity and positive predictive value in detecting active TB with the ICD-10 codes combined with antibiotic prescriptions have been reported [21]. We also missed patients managed in outpatient care setting who were diagnosed with NTM-PD but did not receive any treatments due to their advanced age or due to milder stage of disease among other reasons. In addition, patients who died before the diagnosis could be established may have also led to an underestimation of our results. However, the incidence and prevalence of NTM-PD in our study are in line with other European studies and thus suggest that we did not miss many cases.

## Conclusion

Despite the incremental risk of mortality in NTM-PD patients and financial burden associated with this condition, there is still a critical unmet need for the diagnosis and management of NTM-PD. Only a minority of patients get treated with antibiotics, but many discontinue treatment before the infection can be successfully eradicated. These results underline the high burden associated with NTM-PD and emphasize the need for effective management.

## Supplementary Information

**Additional file 1.** Algorithm used to identify NTM patients (ICD-10 codes and antibiotics combination).

## Data Availability

All data generated or analyzed during this study are included in this published article [and its additional information files]. The data that support the findings of this study are available from [the French Health Insurance (CNAM)] but restrictions apply to the availability of these data, which were used under license for the current study, and so are not publicly available.

## Abbreviations

CCI: Charlson Comorbidity Index
CMUc: Supplementary universal health care coverage
COPD: Chronic obstructive pulmonary disease
HR: Hazard ratio
ICD: International Classification of Diseases
MAC: *Mycobacterium avium* Complex
NTM-PD: Non-tuberculous mycobacteria pulmonary disease
SNDS: Système National des Données de Santé
UK: United Kingdom
