# Supplementary material for: Non-tuberculous mycobacterial pulmonary diseases in France: an 8 years nationwide study
Source: BMC Infect Dis. 2021 Nov 17;21:1165. doi: 10.1186/s12879-021-06825-x (PMC8600813; doi:10.1186/s12879-021-06825-x)
Supplement: Supplementary file 1 — Additional file 1. Algorithm used to identify NTM patients (ICD-10 codes and antibiotics combination). [file 12879_2021_6825_MOESM1_ESM.docx]

Additional files (Methods Part)

# Study populations

## Inclusion criteria

All new cases (adults and children) affiliated to a health insurance scheme of NTM-PD patients newly diagnosed on the period 2010/01/01 through 2017/31/12 AND with no diagnosis, or hospitalizations or treatments of NTM-PD for the last 3 years before the index date were included in the study.

NTM patients were identified through:

1. Hospitalizations with ICD-10 codes specific of NTM infection,
2. Antibiotic drugs associations specific of NTM infection.

### Hospitalizations with ICD-10 codes specific of NTM infection

Patients hospitalized with one of the 3 following ICD-10 codes in MSO (as main or secondary diagnoses):

- A310, « Pulmonary mycobacterial infection”

OR

- A319, “Mycobacterial infections, unspecified”, when associated with the following codes

OR

- A318, “Other mycobacterial infections” when associated with the following codes

The two latter codes A319 and A318 needed to be associated with one of the following respiratory affections:

- - J40 Bronchitis, not specified as acute or chronic.
  - J41 Simple and mucopurulent chronic bronchitis
  - J42 Unspecified chronic bronchitis
  - J43 Emphysema
  - J44 Other chronic obstructive pulmonary disease
  - J47 Bronchiectasis

These codes were searched before the date of the hospital stay where the A318 or A319 were identified.

### Antibiotic drugs associations specific of NTM infection

Outpatient consumptions of drugs were used to select the combinations of treatments that are considered as specific of NTM-PD treatment. Delivery should have been for at least one month because lower duration of treatments can be used for other diseases. Even if the recommended duration of treatment is at least one year, some patients are interrupting the treatment relatively rapidly (after one month).

The followings tables present the antibiotic drugs associations specific of NTM infection.

Table A1. Avium complex (at least one month of treatment)

| First drug | Second drug | Third drug |
| --- | --- | --- |
| Clarithromycin (alone) |  |  |
| Clarithromycin | +**Rifampycinor rifabutin** |  |
| Clarithromycin | +**Rifampycin** | **+Ethambutol** |
| Clarithromycin | +**Ethambutol** |  |
| Azithromycin | +**Rifampycin** |  |
| Azithromycin | +**Rifampycin** | **+Ethambutol** |
| Azithromycin | **+Ethambutol** |  |

Table A2. Xenopi combinations

| First drug | Second drug | Third drug |
| --- | --- | --- |
| Clarithromycin (alone) |  |  |
| Clarithromycin | +**Rifampycin** |  |
| Clarithromycin | +**Rifampycin** | **+Ethambutol** |
| Clarithromycin | +**Ethambutol** |  |
| Moxifloxacin (alone) |  |  |
| Moxifloxacin | +**Rifampycin** |  |
| Moxifloxacin | +**Rifampycin** | **+Ethambutol** |
| Moxifloxacin | **+Ethambutol** |  |
|  | +**Rifampycin** |  |

Table A3. Abcessus combinations

| First drug | Second drug | Third drug |
| --- | --- | --- |
| Clarithromycin | **+ Amikacine (IV or nebulisation)  minimum duration ? 1 WEEK*** |  |
| Azithromycin | **+Amikacin (IV)** |  |
| Clarithromycin | **+Amikacin (IV)** | **One or more drug among the list:**   - - **Cefoxitin**   - **Imipenem**   - **Tigecyclin**   - **Clofazimin**   - **Linezolid** |
| - - Clarithromycin +Amikacin (IV) +Cefoxitin   - Clarithromycin +Amikacin (IV) +Cefoxitin+Imipenem   - Clarithromycin +Amikacin (IV) +Cefoxitin+Imipenem+Tigecyclin   - Clarithromycin +Amikacin (IV) +Cefoxitin+Imipenem+Tigecyclin+Clofazimin   - Clarithromycin +Amikacin (IV) +Cefoxitin+Imipenem+Tigecyclin+Clofazimin+Linezolid   - Clarithromycin +Amikacin (IV) +Cefoxitin+Tigecyclin   - Clarithromycin +Amikacin (IV) +Cefoxitin+Tigecyclin+Clofazimin   - Clarithromycin +Amikacin (IV) +Cefoxitin+Tigecyclin+Clofazimin+Linezolid   - Etc… | | |
| Azithromycin | **+Amikacin (IV)** | **One or more drug among the list:**   - - **Cefoxitin**   - **Imipenem**   - **Tigecyclin**   - **Clofazimin**   - **Linezolid** |
| Examples:   - - Azithromycin +Amikacin (IV) +Cefoxitin   - Azithromycin +Amikacin (IV) +Cefoxitin+Imipenem   - Azithromycin +Amikacin (IV) +Cefoxitin+Imipenem+Tigecyclin   - Azithromycin +Amikacin (IV) +Cefoxitin+Imipenem+Tigecyclin+Clofazimin   - Azithromycin +Amikacin (IV)   - +Cefoxitin+Imipenem+Tigecyclin+Clofazimin+Linezolid   - Azithromycin +Amikacin (IV) +Cefoxitin+Tigecyclin   - Azithromycin +Amikacin (IV) +Cefoxitin+Tigecyclin+Clofazimin   - Azithromycin +Amikacin (IV) +Cefoxitin+Tigecyclin+Clofazimin+Linezolid   - Etc… | | |

### Specific situations when patients are identified with both MSO PMSI and specific drugs

For the following situations, patients were considered as NTM patients.

- A310 (Pulmonary mycobacterial infection) + Isoniazid +Rifampycin+Ethambutol (A310 is enough to be considered as a NTM case).
- A16 (Respiratory tuberculosis, not confirmed bacteriologically or histologically) with Isoniazid + Rifampycin+ Ethambutol or pyrazinamide (RIFATER) or rifabutin or streptomycin with SWITCH to one of the specific treatments after one or two months.
- Treated initially with Isoniazid +Rifampycin+Ethambutol or pyrazinamide (RIFATER) or Isoniazid +Rifampycin (RIFINAH) or rifabutin or streptomycin with SWITCH to upper treatment after one or two months.

## Exclusion criteria

These exclusion criteria codes were applied to all patients except those with A310 (certain diagnosis):

- A15: Respiratory tuberculosis, bacteriologically and histologically confirmed.
- J15.1: Pneumonia due to Pseudomonas
- A16: Respiratory tuberculosis, not confirmed bacteriologically or histologically with Isoniazid +Rifampicin +Ethambutol or pyrazinamide (RIFATER) or rifabutin or streptomycin.

They were identified during hospitalizations, before and around the identification of A310 and specific treatments. Patients who had no hospital stay with a diagnosis of NTM and who had delivery of drugs / combination of drugs not specific of NTM were excluded of the analyses:

- Monotherapy with
  - moxifloxacin
  - clarithromycin
- Polytherapies with:
  - moxifloxacin + ethambutol
  - moxifloxacin + rifampycin
  - clarithromycin + moxifloxacin
  - Azithromycin + moxifloxacin
  - Azithromycin + Clarithromycin
